# The Reparative Effects of Radio Electric Asymmetric Conveyer Technology on Facial Injuries: A Report of Two Cases

**DOI:** 10.7759/cureus.26273

**Published:** 2022-06-24

**Authors:** Vania Fontani, Alessandro Castagna, Salvatore Rinaldi

**Affiliations:** 1 Research Department, Rinaldi Fontani Foundation, Florence, ITA; 2 Department of Regenerative Medicine, Rinaldi Fontani Institute, Florence, ITA

**Keywords:** reac, biostimulation, regenerative medicine treatments, reparative medicine treatments, facial trauma

## Abstract

Facial injuries are often caused by accidental traumas and can be devastating, as their aesthetic outcomes can impact the social relationships and self-esteem of the patient. The reparative processes can be delayed and hindered by the alteration of the endogenous bioelectric activity (EBA) in the damaged tissues, caused by the trauma. In fact, the proper maintenance and generation of EBA is a prerequisite for the cellular health of any tissue and the alteration of EBA determines the inhibition of any cellular repair process, affecting even the cellular differentiation processes. The radio electric asymmetric conveyer (REAC) technology for neurobiological stimulation treatments was designed precisely to restore EBA in both superficial and deep tissues. The two cases of facial trauma presented in this report were treated with the noninvasive treatment of reparative tissue optimization (TO-RPR) applied with REAC technology. The results showed that the REAC TO-RPR treatment can quickly and safely optimize the reparative processes of the tissues, inducing a homogeneous, synchronized, and coordinated recovery, regardless of the type and the aging of the injured tissue and the severity and depth of the lesions.

## Introduction

Facial injuries are frequently encountered in clinical practice and can be caused by various events, often due to accidental traumas. Depending on the traumatic agent, these injuries can be devastating, as they can cause structural lesions of the facial skeleton, the eyes, the airways, and the auditory system, especially external. In addition to the traumatic damage itself, these injuries are often associated with aesthetic and psychological trauma. In particular, the aesthetic results of trauma can affect the patient's social relationships and self-esteem. Hence, it is important to employ therapeutic approaches that can promote the therapeutic processes of repairing the injured tissues, thereby preventing outcomes that can disfigure the aesthetics of the face. Nowadays, one of the therapeutic tools used to treat this type of injury is the noninvasive treatment of reparative tissue optimization (TO-RPR) [1-3], which is enabled by the radio electric asymmetric conveyer (REAC) technology for neurobiological stimulation treatments. In this report, we discuss our experience with two cases of facial trauma from bicycle accidents.

## Case presentation

Case 1

The first case concerns an 85-year-old man. The patient suffered a disastrous bicycle accident with a forward projection and fall on the face, resulting in lacerated bruises on the back of the nose and on the glabella, conspicuous peri glabellar ecchymotic suffusions on the back of the nose and affecting the periorbital area, particularly suborbital, as well as edema of the nose and of the perioral area. Promptly rescued, he was given a first dressing and application of suture plasters on the wound of the bridge of the nose.

**Figure 1:**
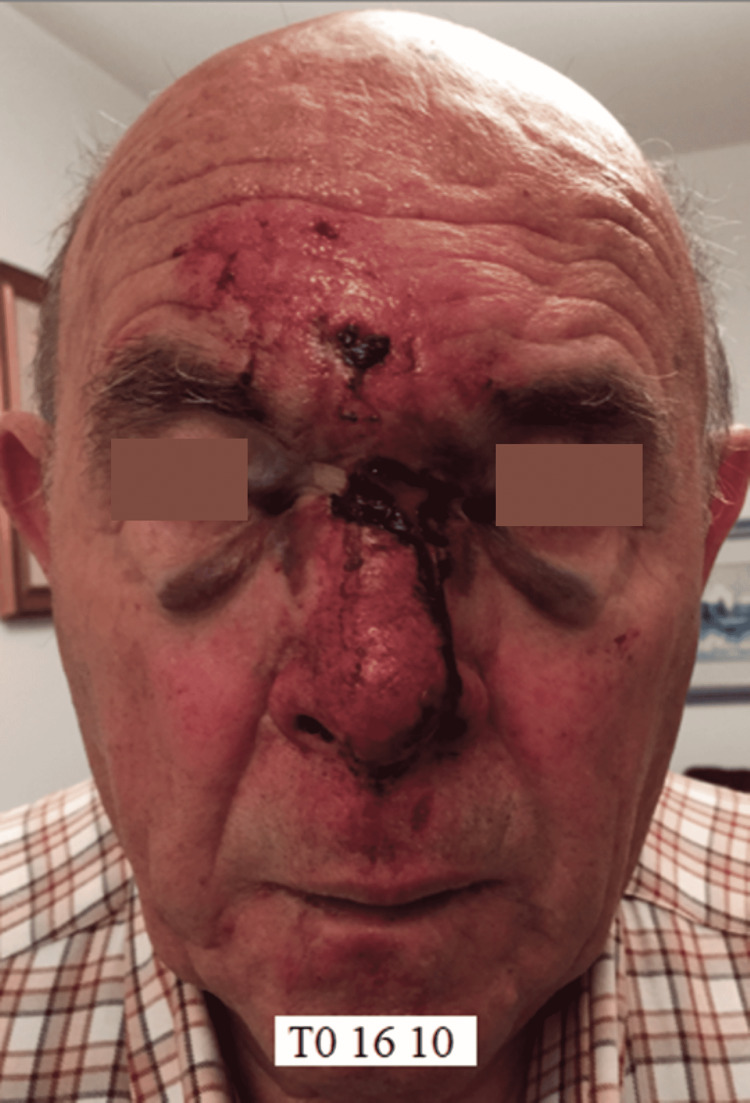
Pre-treatment facial trauma at T0

On October 16 (T0), the day after the trauma, the patient presented to us for observation (Figure 1) and was prescribed a cycle of 18 sessions of REAC TO-RPR treatment.

A REAC TO-RPR treatment session can last for 15 to 30 minutes; in this case, 30-minute treatment sessions were used. The REAC TO-RPR treatment parameters are preprogrammed and cannot be changed by the operator. The cycle of 18 sessions of REAC TO-RPR 30 was distributed over five consecutive days. The minimum interval between each session was at least one hour for a maximum of four treatment sessions per day.

**Figure 2:**
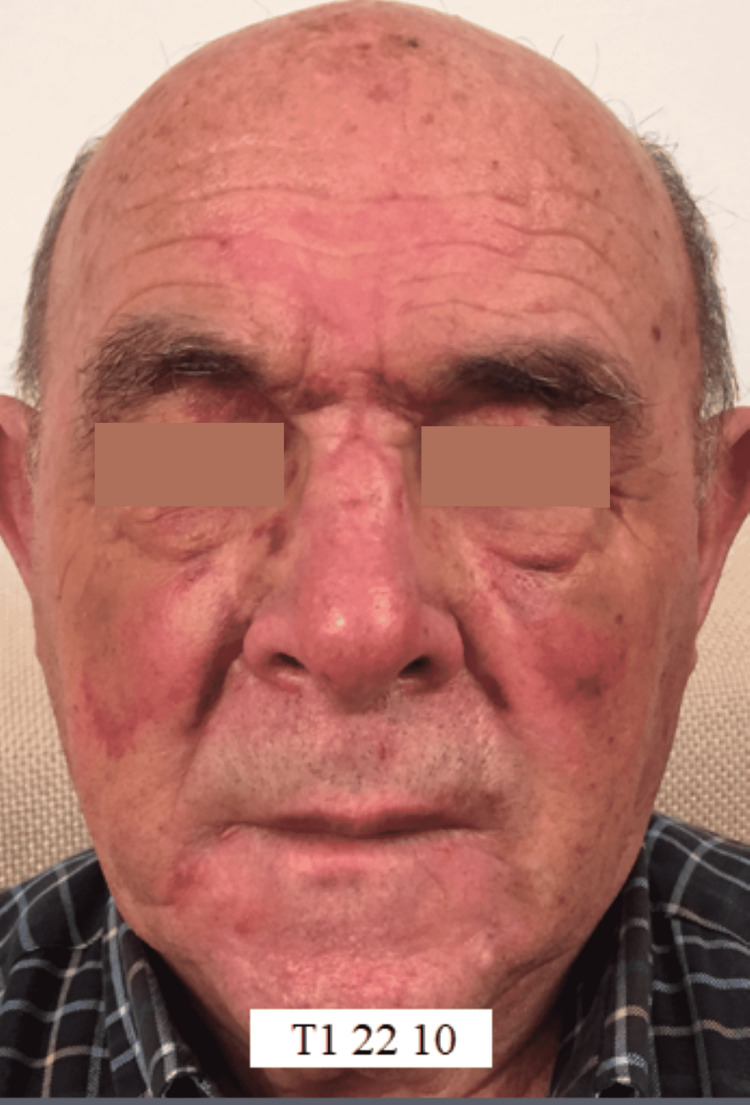
Post-treatment facial trauma at T1 At T1, the patient shows rapid improvement induced by the REAC TO-RPR treatment both in lacerated bruised wounds, ecchymotic suffusions, and facial edema, almost causing their disappearance REAC TO-RPR: radio electric asymmetric conveyer treatment for reparative tissue optimization

During the five days of treatment, rapid and progressive improvement was observed both in the lacerated bruised wounds and ecchymotic suffusions, resulting in the almost complete disappearance of all lesions and facial edema (Figure 2).

**Figure 3:**
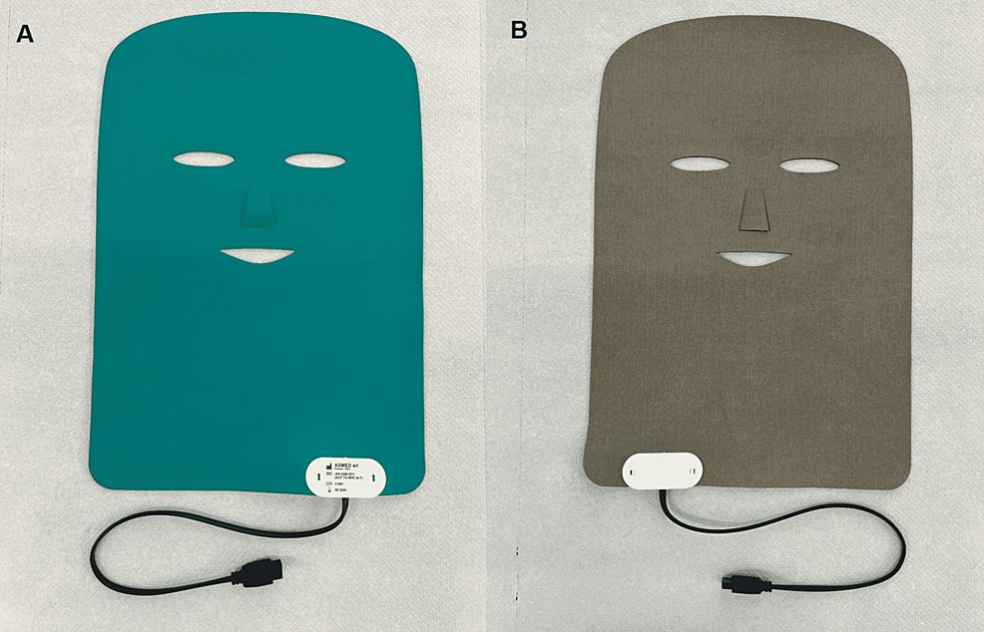
ACP specially designed for facial reparative treatments Panel A shows the external side, while B represents the internal side. The internal side (B) consists of a special superconducting fabric. The white box connected to the ACP contains a microprocessor, which, once connected to the BENE 110 device through the cable, allows for optimizing the performance of the treatment ACP: asymmetric conveying probe

The REAC TO-RPR treatment is administered by placing an asymmetric conveying probe (ACP) on the area to be treated (Figures 3A, 3B).

The ACP is kept on the area by means of a tubular elastic gauze. The ACP is connected to the REAC device, BENE Model 110 (therapeutic electromedical equipment for neurobiological stimulation CE 1282; ASMED SRL, Florence, Italy).

Case 2

**Figure 4:**
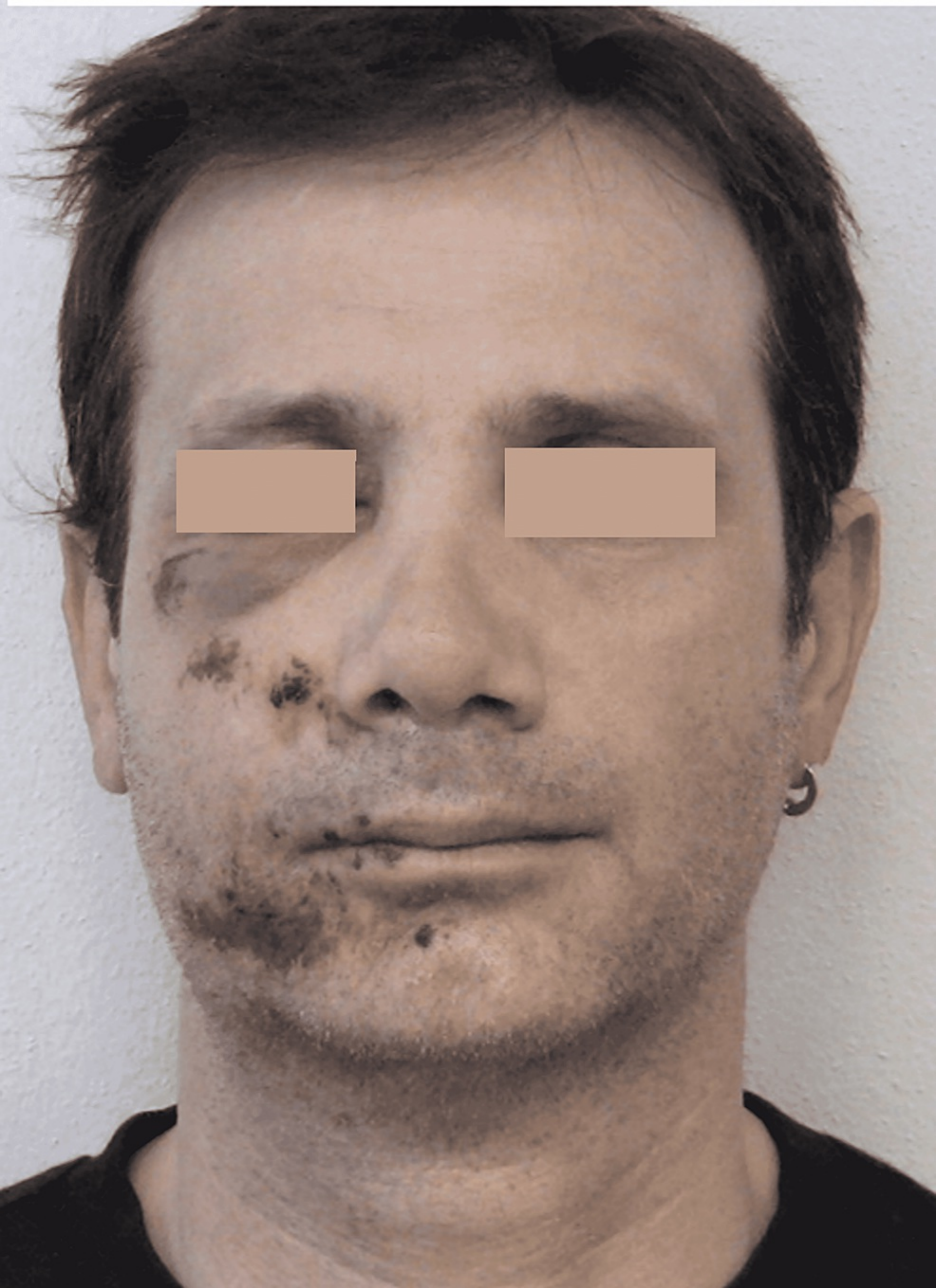
Pre-treatment facial trauma

The second case concerns a 45-year-old man who fell off his bicycle during a workout. The fall caused lacerated bruises on the face and multiple deep excoriations on the right half-part, conspicuous periocular ecchymotic suffusions, particularly suborbital, and edema of the right cheek and the perioral area. A few hours after the trauma, the patient presented to our facility for observation (Figure 4).

After two sessions of TO-RPR 30 treatment, the patient already showed a very rapid and progressive improvement of the bruised laceration and ecchymotic suffusions.

**Figure 5:**
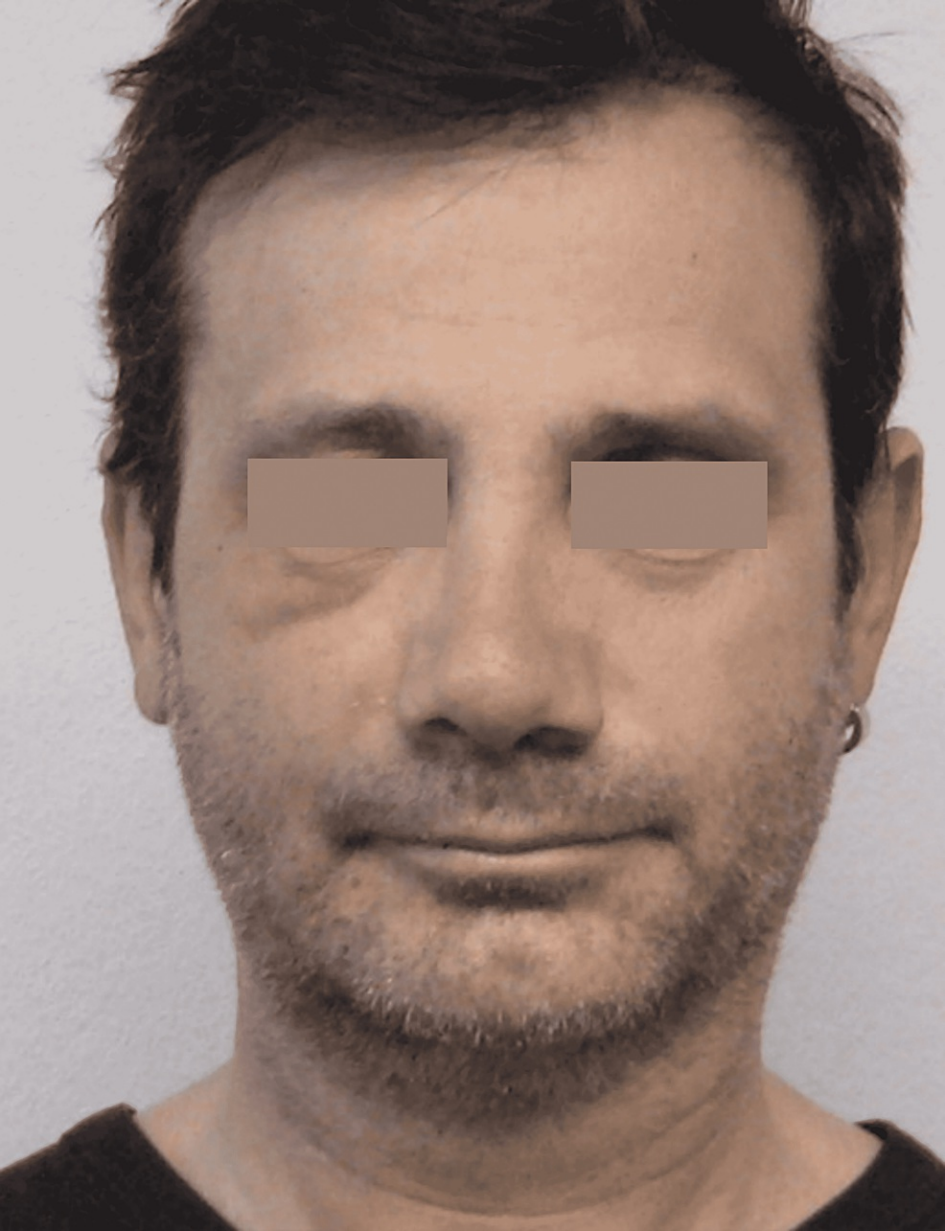
Post-traumatic facial injuries after two sessions of REAC TO-RPR treatment of 30 minutes administered on the same day (control after 2 days) REAC TO-RPR: radio electric asymmetric conveyer treatment for reparative tissue optimization

Noting the very rapid improvement, after the second session of REAC TO-RPR 30, it was decided to suspend the treatment and analyze the evolution after two days. At the check-up (Figure 5), as the patient showed almost total healing of the facial lesions, no more REAC TO-RPR session was administered.

## Discussion

All traumas are unwelcome events, and those affecting the face are especially so; facial injuries post-trauma can cause severe aesthetic damage that can persist if not treated properly. Consequently, it is very important that the patient receives therapeutic treatment to minimize the effects of the trauma suffered and its impact on the patient’s physical and psychological health.

One of the major obstacles that prevent or delay the reparative processes is the fact that traumas can lead to an alteration of the proper endogenous bioelectric activity [4] (EBA) in the injured tissues. Proper maintenance and generation of EBA is a prerequisite for the cellular health of any tissue [4]. The alteration of EBA, induced by injuries of various origins, determines the inhibition of any cellular repair process, affecting even the cellular differentiation processes. The REAC technology was designed with the precise aim of restoring EBA in both superficial and deep tissues, to the extent of epigenetically determining direct cellular reprogramming with specific treatment protocols [5-7].

Since each tissue has different characteristics and can be damaged by different causes - physical, infectious, or degenerative - over the years, several protocols have been studied to optimize the therapeutic results [8-11]. In this regard, the effects of the REAC TO-RPR treatment have shown how the restoration of EBA and the recovery of the tissues affected by lacerated bruises have a homogeneous, synchronized, and coordinated course, irrespective of the type of tissue and the severity and depth of the lesions.

Moreover, the results of the REAC TO-RPR treatment do not seem to be conditioned by the age of the patient and therefore by the aging of the tissues. This is particularly evident in the first case, which involved a patient aged 85 years (Figures 1, 2). In the second case described, the reparative effectiveness of the TO-RPR treatment proved to be particularly rapid (Figures 4, 5), probably because the lesions were less destructive in nature.

It is interesting to highlight that the reparative effects induced by the REAC TO-RPR treatment prevented the formation of scarring, and this finding has already been demonstrated by other previously published studies [1-3].

## Conclusions

Based on our findings, the REAC TO-RPR neurobiological modulation treatment can optimize the reparative processes by remodeling the bioelectrical alterations of the injured tissues. The REAC TO-RPR treatment can quickly and safely induce the recovery of the lesions and prevent unaesthetic outcomes.
